# Gut brain interaction theory reveals gut microbiota mediated neurogenesis and traditional Chinese medicine research strategies

**DOI:** 10.3389/fcimb.2022.1072341

**Published:** 2022-12-08

**Authors:** Chenxi Zhang, Peng Xue, Haiyan Zhang, Chenxi Tan, Shiyao Zhao, Xudong Li, Lihui Sun, Huihui Zheng, Jun Wang, Baoling Zhang, Weiya Lang

**Affiliations:** ^1^ Basic Medical Science College, Qiqihar Medical University, Qiqihar, China; ^2^ Medical School of Nantong University, Nantong University, Nantong, China; ^3^ Department of Infection Control, The Second Affiliated Hospital of Qiqihar Medical University, Qiqihar, China; ^4^ Department of Nuclear Medicine, The Third Affiliated Hospital of Qiqihar Medical University, Qiqihar, China; ^5^ Department of Breast Surgery, Harbin Medical University Cancer Hospital, Harbin, China; ^6^ The Academic Affairs Office, Qiqihar Medical University, Qiqihar, China; ^7^ Department of Operating Room, Qiqihar First Hospital, Qiqihar, China

**Keywords:** neurogenesis, gut microbiota, traditional Chinese medicine, microbiota-gut-brain axis (MGB axis), neural stem cell (NSC)

## Abstract

Adult neurogenesis is the process of differentiation of neural stem cells (NSCs) into neurons and glial cells in certain areas of the adult brain. Defects in neurogenesis can lead to neurodegenerative diseases, mental disorders, and other maladies. This process is directionally regulated by transcription factors, the Wnt and Notch pathway, the extracellular matrix, and various growth factors. External factors like stress, physical exercise, diet, medications, etc., affect neurogenesis and the gut microbiota. The gut microbiota may affect NSCs through vagal, immune and chemical pathways, and other pathways. Traditional Chinese medicine (TCM) has been proven to affect NSCs proliferation and differentiation and can regulate the abundance and metabolites produced by intestinal microorganisms. However, the underlying mechanisms by which these factors regulate neurogenesis through the gut microbiota are not fully understood. In this review, we describe the recent evidence on the role of the gut microbiota in neurogenesis. Moreover, we hypothesize on the characteristics of the microbiota-gut-brain axis based on bacterial phyla, including microbiota’s metabolites, and neuronal and immune pathways while providing an outlook on TCM’s potential effects on adult neurogenesis by regulating gut microbiota.

## Introduction

The brain development and normal brain function are closely related to neuron and glial cells cytogenesis and activation. This process is also closely related to neural stem cells (NSCs) differentiation. NSCs are a kind of blast cells with multi-directional differentiation potential and self-renewal ability, which can proliferate and differentiate into neurons, astrocytes, and oligodendrocytes, either during embryonic development or in the adult brain (Altman and Das, 1965; Bottes et al., 2021), although NSCs remain dormant for a long time after the embryonic stage. In the adult brain, NSCs are found in the ventricular-subventricular zone (V-SVZ) lining the lateral ventricles, where olfactory bulb neurons, astrocytes, and oligodendrocytes are generated, and the subgranular zone (SGZ) of the dentate gyrus (DG) in the hippocampus, where neurons are mainly generated (Ming and Song, 2011; Song et al., 2016; Mizrak et al., 2019). In addition, the hypothalamus may be another neurogenic region in the brain: dorsal α1 region, dorsal α2 region, and adjacent median eminence (Andreotti et al., 2019). NSCs proliferation and differentiation is a finely controlled and complex process. In V-SVZ, the embryonic radial glia can differentiate into NSCs of the V-SVZ (B1 cells). As adults, B1 cells undergo symmetric division to complete cell self-renewal or undergo asymmetric division to form intermediate progenitors (C cells), which further generate neuroblasts (A cells) (Ponti et al., 2013; Fuentealba et al., 2015; Obernier et al., 2018). In the SGZ, these NSCs are known as radial glia-like cells (or type 1 cells); when activated, they produce actively proliferating neural progenitor cells (NPCs or type 2 cells), which proliferate and produce neuroblasts (type 3 cells), which exit the cell cycle and differentiate into neurons (Urban et al., 2019; Harris et al., 2021). Elucidating the regulatory mechanism of NSCs proliferation and differentiation is crucial for understanding nervous system development, nerve repair, and cell transplantation for the treatment of nervous system diseases. Current studies have shown that the main factor for self-renewal, differentiation, and maintenance of NSCs is genetic and depends on the co-integration of multiple cellular signaling systems in the microenvironment, including the Wnt, Notch, Sonic hedgehog (SHH) pathways, etc.

Currently, the gut microbiota has been shown to play an important role in regulating neuronal and glial function, interfering with neuron and glial cytogenesis and activation through the microbiota-gut-brain (MGB) axis, which is mainly composed of the enteric nervous system, central nervous system (CNS), and immune system (Cryan et al., 2019; Gershon and Margolis, 2021). The gut microbiota establishes two-way communication with the brain through its metabolites, which cross the blood-brain barrier (BBB), pass through the vagus nerve, or induce peripheral immunity (Morais et al., 2021). Therefore, changes in the structure of the gut microbiota may affect NSCs differentiation and neurogenesis.

Traditional Chinese medicine (TCM) is a medical system with a long history and is widely used in the prevention and treatment of nervous system diseases and regulation of body homeostasis. The active ingredients of TCM play an important role in regulating NSCs differentiation, and neuron and glia generation and activation (Qin et al., 2017; Wang X. F. et al., 2021). Many reports have indicated that TCM can impact the composition and metabolism of the gut microbiota (Feng et al., 2019). Gut microbiota can release signaling substances that promote the development and maintenance of host digestive, immune, metabolic, and neurobiological functions, and these signaling substances also be regulated by drug. TCM has shown great potential in regulating the gut microbiota to influence NSCs. This review highlights several advanced mechanisms of signaling pathways in neurogenesis and systematically discusses potential TCM strategies to regulate NSCs through the gut microbiota.

## Signaling pathways involved in NSCs proliferation and differentiation

**Figure 1:**
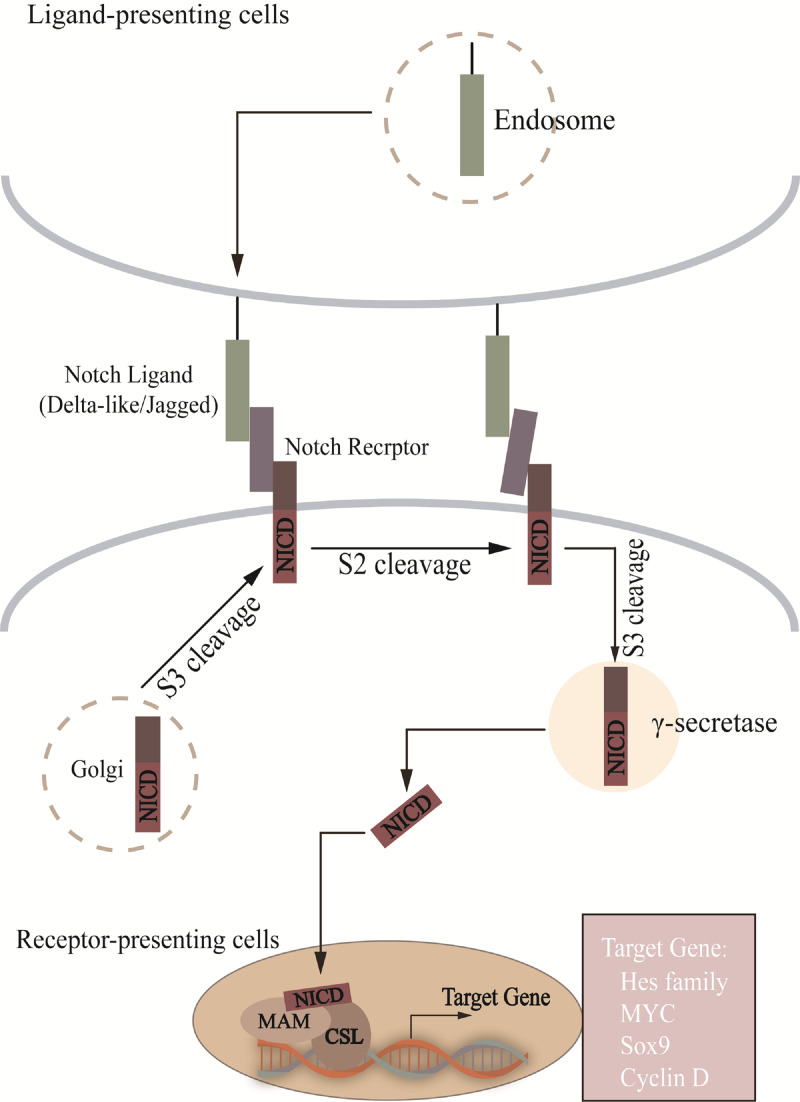
In Notch signal pathway, Notch is cut into heterodimer (S1 cleavage) in Golgi and transferred to cell membrane. S2 site is exposed after ligand-receptor interaction, which passes through AMAD metalloproteases and γ-secretase enzyme processing, translocation to the nucleus to combine with CSL, recruitment of MAM, and activation of target gene transcription. NICD, notch intracellular structural domain; MAM, mastermind; CSL, CBF1/RBP-J/Su(H)/Lag-1.

**Figure 2:**
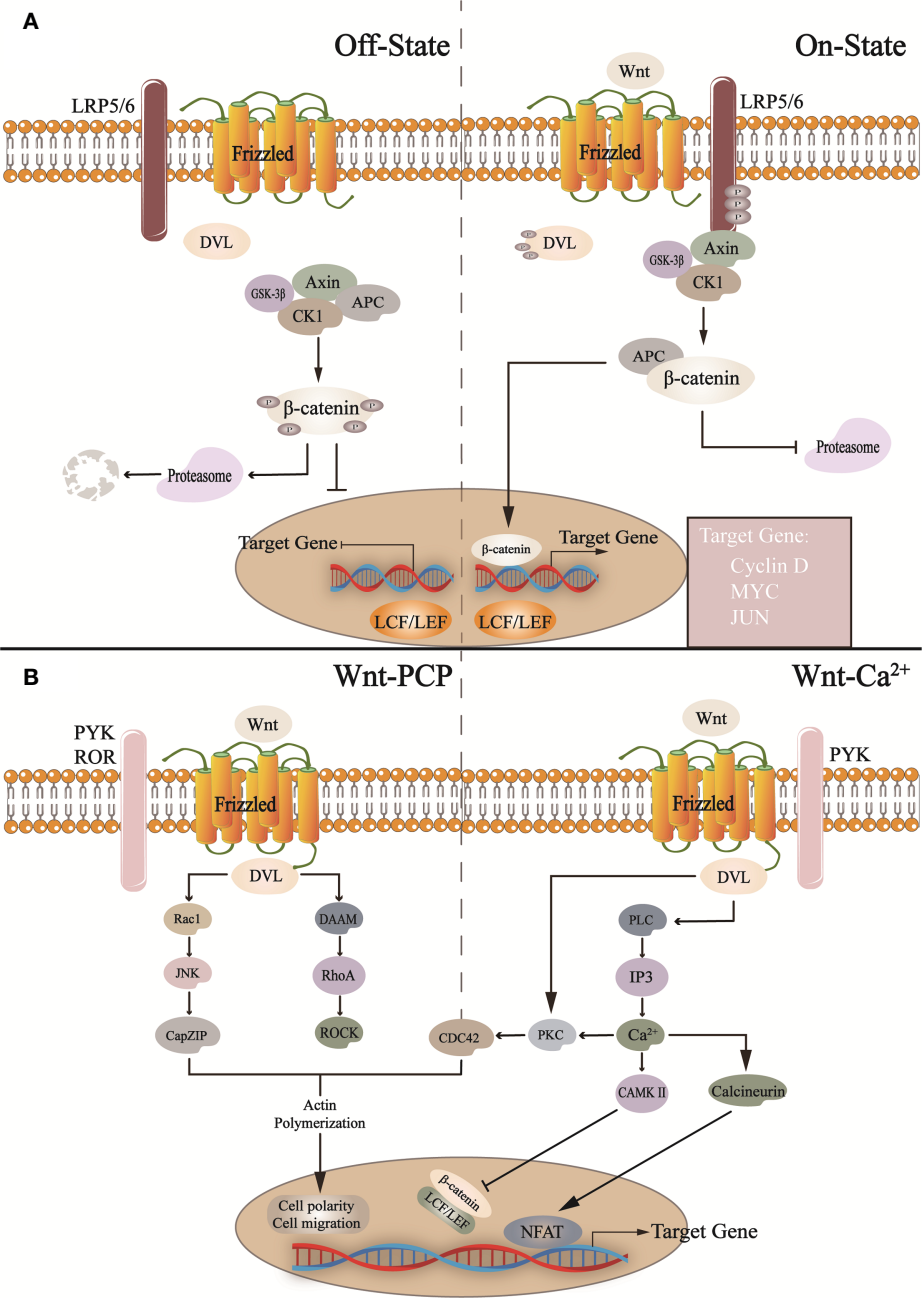
In the canonical Wnt pathway **(A)**: In the absence of Wnt activation, the cytoplasmic β-catenin is phosphorylated by the destruction complex (Axin, CK1, GSK-3β, APC) and then degraded by proteasome after a series of reactions. When Wnt binds to Frizzled and LRP5/6 receptors, it activates DVL, inactive GSK-3β, and inhibits proteasome degradation β-catenin, and β-catenin translocates into the nucleus and binds with LCF/LEF to regulate target gene transcription. In the non-canonical Wnt pathway. CK1, casein kinase 1; GSK-3b, glycogen synthase kinase 3β; APC, adenomatous polyposis coli; LPR5/6, low-density lipoprotein-related receptors 5 and 6; DVL, Dishevelled. **(B)** In the Wnt/PCP pathway, Wnt binds to other receptors (such as ROR, RYK, etc.), activates DVL, and promotes downstream pathway transduction, such as Roc1, JNK, etc. This is related to cell migration and cell polarity. In addition, in the Wnt/Ca^2+^ pathway, it activates phospholipase C (PLC), triggers intracellular Ca^2+^ release, and regulates NFAT and β-catenin, which regulates the expression of related genes. Roc1, regulator of cullins 1; JNK, c-Jun N-terminal kinase; CapZIP, CapZ-interacting protein; DAAM, Dishevelled associated activator of morphogenesis; RhoA, Ras homolog gene family member A; ROCK, Rho-associated, coiled-coil containing kinases; PLC, phospholipase C; IP3, inositol 1,4,5-trisphosphate; PKC, protein kinase C; CAMKII, calcium/calmodulin-dependent protein kinase II; NFAT, nuclear factor of activated T-cells; PYK, RYK receptor-like tyrosine kinase; ROR, receptor tyrosine kinase-like orphan receptor.

**Figure 3:**
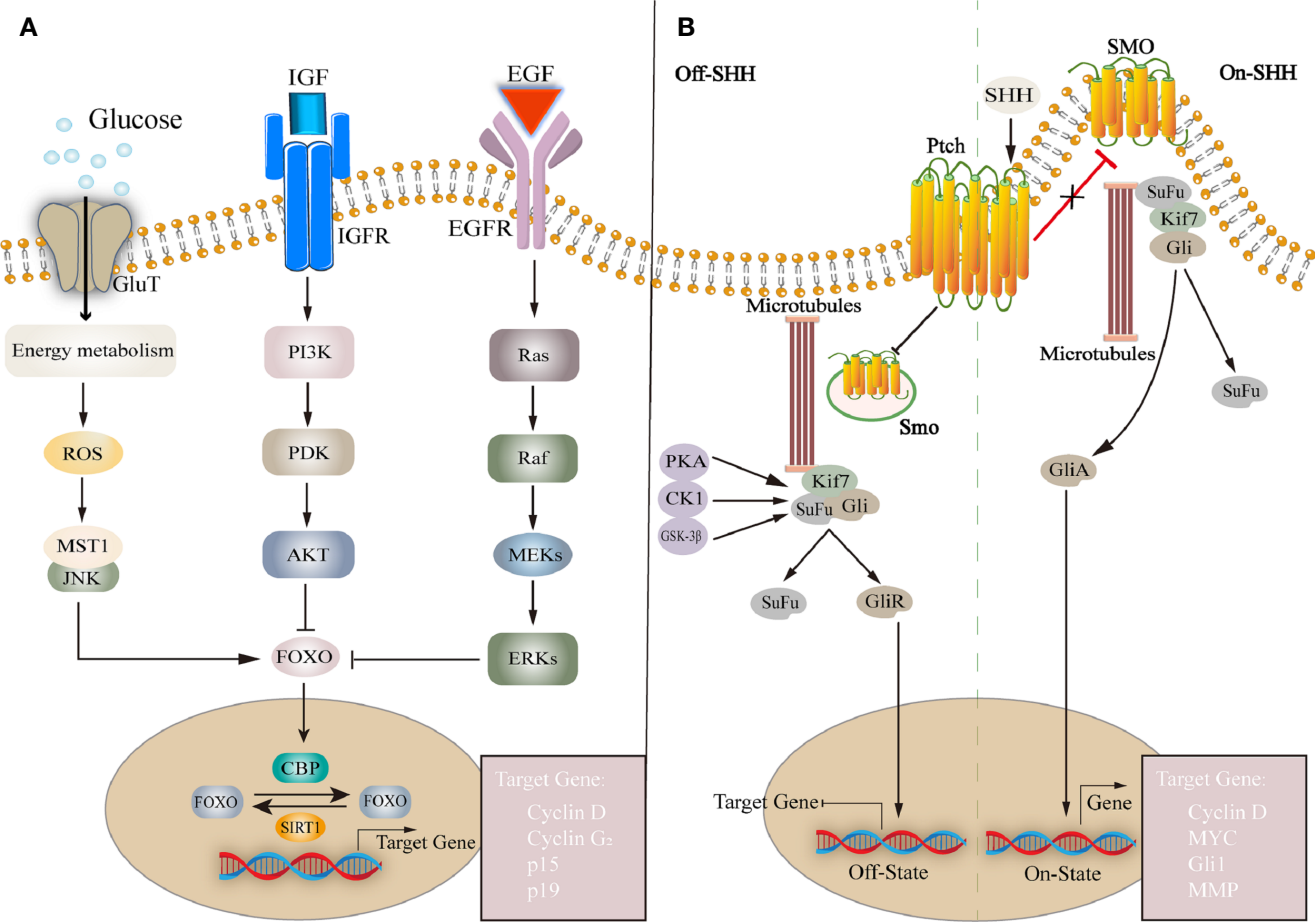
**(A)** FOXO family is a group of transcription factors that regulate the expression of downstream genes and affect cell proliferation, cell cycle, etc. FOXO is affected by many factors, such as insulin or IGF, EGF, glucose, etc. Regulated transcription by regulating histone acetyltransferases CBP and deacetylase IGF, insulin-like growth factor; EGF, epidermal growth factor; ROS, reactive oxygen species; MST1, mammalian sterile 20-like kinase 1; PI3K, phosphoinositide 3-kinase; PDK, phosphoinositide-dependent protein kinase; AKT, protein kinase B; FOXO, forkhead box O; SIRT1, silencing information regulator 2 related enzyme 1. **(B)** SHH pathway: Without SHH, Ptch inhibits Smo activity, Gli is phosphorylated by PKA, forming a repressor GliR, which is translocated to the nucleus to inhibit target gene transcription. Extracellular SHH binds to Ptch, and Smo is no longer inhibited. forming a repressor GliA, translocated to the nucleus and activation of target gene expression. Ptch, Patched; Smo, Smoothened; SuFu, suppressor of fused; Kif7, kinesin family member 7; Gli, Glioma-associated oncogene.

The proliferation, differentiation and migration of NSCs in the neurogenic niches maintain neurogenesis by interacting with the glial cells, extracellular matrix and microenvironment of the niches. **Table 1** summarizes the effects of cellular and microenvironmental components of different niches on NSCs. Changes in the cell, the extracellular matrix and microenvironmental components affect the function of NSCs, that is, by changing external factors and affecting relevant signal pathways, such as Notch, Wnt, SHH, FOXO, and other pathways, proteins or factors (**Figures 1**–**3**), and influence neurogenesis. Thus, changes in the abundance of the gut microbiota may be able to influence adult neurogenesis by altering the cells, the extracellular matrix and components of the microenvironment in certain ways.

**Table 1:** The Alternations and Influences of Niche Factors in Different Regions on Neurogenesis.

Neurogenic niche	Source in the niche	Niche factor	Neurogenic phenotype	Reference
V-SVZ	Astrocytes	Dlk1	Cell proliferation, neuronal differentiation	(Ferron et al., 2011)
		Jagged1	Cell proliferation	(Wang et al., 2009)
		SHH	Cell proliferation, neuronal differentiation, cell migration	(Palma et al., 2005) (Tong et al., 2015) (Angot et al., 2008)
		Wnts	Cell proliferation, neuronal differentiation	(Yu et al., 2006)
		BMPs	Cell proliferation, neuronal differentiation	(Bond et al., 2012)
	Microglia	Inflammatory factor	Cell proliferation, neuronal differentiation	(Willis et al., 2022) (Shigemoto-Mogami et al., 2014)
		IGF-α	Cell migration	(Hurtado-Chong et al., 2009)
	Ependymal cell	BNDF	Cell proliferation, neuronal differentiation, cell migration	(Ribeiro and Xapelli, 2021)
		MMP12	NSC quiescence, cell proliferation	(Shan et al., 2018)
		CCN1	Cell proliferation, neuronal differentiation	(Wu et al., 2020)
	Choroid plexus	miR-204	Cell proliferation, neuronal differentiation	(Lepko et al., 2019)
		OTX2 homeoprotein	Cell migration	(Planques et al., 2019)
		SHH	Cell proliferation, neuronal differentiation	(Kinoshita et al., 2022)
		CSF	Cell migration	(Sawamoto et al., 2006)
	Endothelial cell	VEGF	NSC activation, cell migration	(Wittko et al., 2009) (Han et al., 2015)
		Angiopoietin-1	Cell proliferation, neuronal differentiation	(Rosa et al., 2010)
		Laminin	Cell proliferation	(Rosa et al., 2016)
		NT-3	NSC quiescence, cell proliferation, neuronal differentiation	(Silva-Vargas and Doetsch, 2014)
	Others	Fibrinogen	Neuronal differentiation	(Pous et al., 2020)
		Ghrelin	Cell proliferation, neuronal differentiation, cell migration	(Li et al., 2014)
		GABA	Cell migration	(Hsieh and Puche, 2015)
		5-HT	Cell proliferation	(Banasr et al., 2004)
		Dopamine	Cell proliferation, neuronal differentiation	(O’Keeffe et al., 2009)
SGZ	Astrocytes	Jagged1	Cell proliferation, neuronal differentiation	(Wilhelmsson et al., 2012)
		BDNF	Cell proliferation, neuronal differentiation	(Waterhouse et al., 2012)
		Thrombospondins	Synaptogenesis	(Christopherson et al., 2005)
	Microglia	Inflammatory factor	Cell proliferation	(Mcpherson et al., 2011)
		Phagocytosis	Cell survival	(Sierra et al., 2010)
		miR-146a-5p	Cell proliferation, neuronal differentiation, cell migration	(Fan et al., 2022)
	Endothelial cell	Lactate	Cell proliferation, neuronal differentiation	(Wang et al., 2019)
		VEGF	Cell proliferation	(Jin et al., 2002)
	Granule cell	Ephrin-B3	NSC quiescence	(Dong et al., 2019)
		Noggin	Cell proliferation, neuronal differentiation	(Bonaguidi et al., 2008)
	Others	Melatonin	Cell proliferation	(Fredrich et al., 2017)
		GABA	NSC quiescence	(Bao et al., 2017)
		SHH	Cell proliferation, cell migration	(Gonzalez-Reyes et al., 2019)

BMP, bone morphogenetic protein; MMP12, matrix metallopeptidase 12; CCN1, cellular communication network factor 1; NT-3, neurotrophin-3.

During brain development, both in embryos and adult animals, the Notch pathway is involved in NSCs regulation at various developmental stages, maintaining the number of NSCs in the brain in a dynamic balance, which is crucial for the development and maturation of the CNS (**Figure 1**) (Androutsellis-Theotokis et al., 2006; Mizutani et al., 2007; Imayoshi et al., 2013). The Notch pathway consists of Notch receptors (Notch1-4) and ligands such as Delta-like ligands and Jagged (Hori et al., 2013). The Notch receptor is activated by binding with ligands from adjacent cells. Receptor-ligand binding is followed by the release of the Notch intracellular structural domain (NICD) and Nβ peptide in the presence of ADAM metalloproteinase and γ-secretase. NICD enters the nucleus and is recognized by the transcriptional coactivator Mastermind-like proteins after binding to the DNA binding protein CSL (CBF1/RBP-J/Su(H)/Lag-1) and activates transcription (Kopan and Ilagan, 2009). Then the complex induces inhibitory transcription factors such as *Hes*, *Cyclin D*. We hypothesize that changes in the abundance of gut microbiota may affect the expression of Notch ligands and receptors and, to some extent, the function of NSCs and neurogenesis. It was found that after lead (Pb) exposure the intestinal permeability of mice was increased and the abundance of gut microbiota, such as *Bacteroides*, *Lactobacillus*, etc. was altered. The expression of Notch1, RBP-J, Hes1 and Hes2 was also upregulated in the DG and olfactory bulb (Sun et al., 2022). This suggests that after the intervention of TCM, it may affect the abundance of gut microbiota, thereby affecting Notch pathway, which in turn affects NSCs differentiation.

The Wnt pathway widely regulates the maintenance and differentiation of various stem cells *in vivo*. Wnt pathway dysregulation is an important cause of CNS diseases, such as Alzheimer’s disease (AD), Parkinson’s disease (PD), and spinal cord injury (Inestrosa and Arenas, 2010; Garcia-Velazquez and Arias, 2017). The Wnt pathway is divided into canonical Wnt/β-catenin and non-canonical Wnt/PCP and Wnt/Ca^2+^ pathways (**Figure 2**) (Lie et al., 2005; Butler and Wallingford, 2017). The canonical Wnt/β-catenin signaling pathway is mainly involved in NSCs proliferation and differentiation. Wnt is secreted outside the cell through covalent lipid modification in the endoplasmic reticulum (Clevers et al., 2014; Mehta et al., 2021). In the absence of extracellular Wnt ligands from the canonical pathway, β-catenin is degraded by a complex composed of glycogen synthase kinase 3β (GSK-3β) and casein kinase 1(CK1), Axin, etc., and the expression of Wnt target genes is suppressed (Gao et al., 2021). When Wnt ligands bind to Frizzled and Low-density Lipoprotein-related Receptors 5 and 6, they activate downstream signals, reducing β-catenin degradation. After nuclear transfer, the complex binds to the nuclear transcription factor lymphoid enhancer factor/T cell factor complex to induce target genes’ transcription (Macdonald and He, 2012; Hua et al., 2018) and influence NSCs proliferation by stimulating cell cycle-related factors, such as *C-myc*, *Cyclin-D* (Alvarez-Palomo et al., 2021; Liu B. C. et al., 2021; Wang et al., 2016a). Similarly, the lack of gut microbiota down-regulates the expression of neural genes, and gut microbiota metabolites timely save the process, especially affecting Wnt pathway (Rea et al., 2022).

SHH pathway also plays an important role in neurogenesis. The mammalian hedgehog family has three homologous genes *Shh*, *Dhh*, and *Ihh*, encoding hedgehog ligands, and two cell membrane surface molecular receptors Patched (Ptch) and Smoothened (Smo). Ptch negatively regulates protein kinase A (PKA) signaling whereas Smo is a necessary receptor for SHH signal transmission. When the Hedgehog ligand binds to Ptch, its inhibition of Smo stops, and Smo is activated to enter the cell and activate the downstream glioma-associated oncogene (Gli) family (Carballo et al., 2018). Without SHH ligand, Ptch is restricted to the base of the primary cilium and prevents Smo activity, PKA, GSK-3β, and the Gli repressor produced after phosphorylated CK1 enters the nucleus to inhibit the transcription of SHH target genes (Rana et al., 2021), such as *CyclinD*, *Nmyc*, *Bmi1*, *Hey2*, etc. Among them, Gli1 causes NSCs cycle arrest, reduces cell proliferation, and induces NSCs apoptosis. On the contrary, Gli1 inhibition leads to early maturation of NSCs derived oligodendrocytes *in vitro* (Galvin et al., 2008; Namchaiw et al., 2019). In the presence of SHH ligands, SHH and Ptch binding alleviated Smo inhibition and moved to the top of primary cilium, inducing Gli protein to separate from Kinesin family member 7 and Suppressor of Fused, and enter the nucleus to initiate transcription of target genes (Ingham et al., 2011; Briscoe and Therond, 2013). Long-term SHH activation can lead to the accumulation of quiescent neural stem cells in the V-SVZ, while the number of activated neural stem cells transiently increases and later decreases. Taurine, a metabolite of the gut microbiota, plays a unique role in participating in the SHH pathway. It intervenes to regulate the expression of SHH in NPCs and promotes cell proliferation and improves mitochondrial function (Ramos-Mandujano et al., 2014).

## Regulation of neurogenesis by gut microbiota

**Figure 4:**
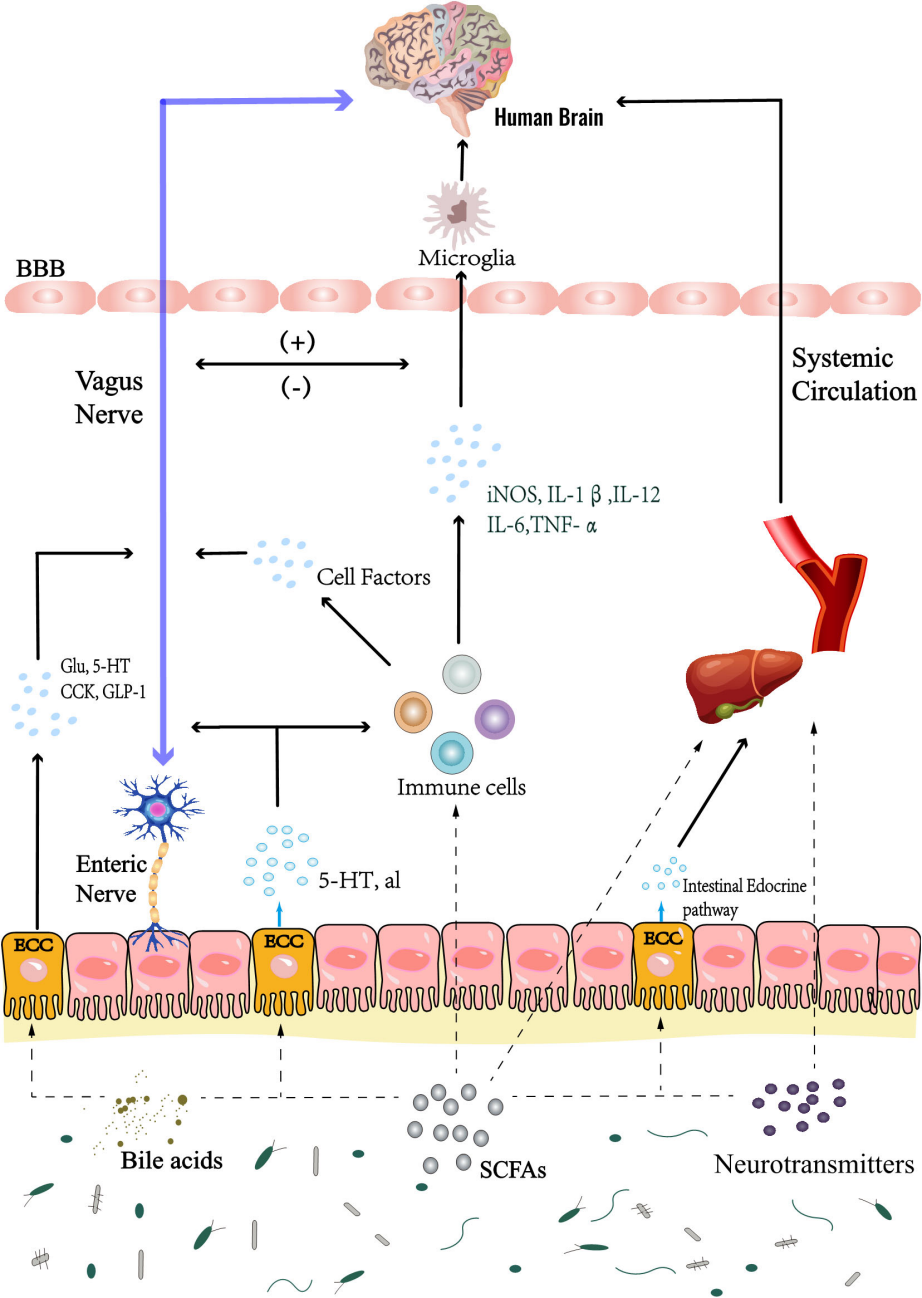
Gut microbiota mainly interacts with the brain through Chemical, Neuronal and Immune pathways. Metabolites of gut microbiota, such as SCFAs, neurotransmitters, bile acids, etc., directly affect the body and brain activities; In addition, the microbiota can also play this role by regulating the activities of enteroendocrine cells, such as ECCs. In the immune pathway, on the one hand, inflammatory factors can change the permeability of blood brain barrier and affect brain activity, these inflammatory factors also affect the brain resident immune cells; On the other hand, it can also affect brain inflammation through cellular immune pathway, which is through intestinal immune cells; Microbial metabolites also regulate the activities of brain resident immune cells. At the same time, inflammatory stimulation can also stimulate the vagus nerve, which also regulates brain activities, such as triggering the activation of HPA axis, pain conduction, etc. In addition, microbial metabolites and secretion of enteroendocrine cells directly affect the vagus nerve, or stimulate the intestinal nervous system to indirectly affect the vagus nerve, and establish a neural pathway to interact with the brain. SCFAs, short chain fatty acids. HPA axis, hypothalamic-pituitary-adrenal axis. ECCs, enterochromaffin cells. BBB, blood-brain barrier; Glu, glutamic acid; 5-HT, 5-hydroxytryptamine; CCK, cholecystokinin; GLP-1, glucagon-like peptide-1.

Gut microbiota is a huge microbial community that inhabits the inner surface of the intestinal mucosa and the intestinal cavity. It is composed of bacteria, viruses, and fungi. Substances produced by the microbiota-metabolism mainly crosstalk between the brain and the gut *via* the MGB axis (Mayer et al., 2022). There is also evidence that the gut microbiota plays a role in influencing NSCs activities (Ogbonnaya et al., 2015). A high sugar diet decreases short chain fatty acids (SCFAs) *in vivo*, causes BBB damage, and decreases the number of NSCs and mature neurons. An effect effectively reversed after SCFAs intervention (Tang C. F. et al., 2022). In addition, butyrate production increased in aseptic mice transplanted with the gut microbiota of aged mice, which stimulated an increase in the number of hippocampal neurons (Kundu et al., 2019). In this section, we will introduce in detail the mechanism by which the gut microbiota may affect the differentiation of NSCs from the MGB axis (**Figure 4** and **Table 2**).

**Table 2:** Effects of Different Phylum Gut Microbiota on Central Nervous System of Host.

Phylum	Bacteria	Channel	Impact on host	Reference
*Bacteroidetes*	*Bacteroides fragilis*	Immune pathway	Inhibited CNS inflammatory demyelination by Polysaccharide A	(Erturk-Hasdemir et al., 2021)
*Firmicutes*	*Lactobacillus rhamnosus*	Neuro-immunoendocrine	Affected the peripheral and central immune system, anti-depression and anti-anxiety	(Liu Y. et al., 2021)
	*Lactobacillus reuteri*	Vagus nerve	Increased IL-6, decrease the expression of synaptic proteins, and affected depression and anxiety	(Wang S. et al., 2020)
	*Clostridium butyricum*	Chemical pathways	Stimulated intestinal GLP-1 secretion, activated brain GLP-1R, increased 5-HT, and up regulated BDNF expression, anti-anxiety	(Sun et al., 2018)
*Proteobacteria*	*Bacillus coli*	Chemical pathways	Produced vitamin K, promoted neuronal differentiation	(Bryant and Bentley, 1976) (Sakane et al., 2017)
*Actinobacteria*	*Bifidobacterium longum, Bifidobacterium bifidum*	N/A	Improved hippocampal synaptic plasticity and neuroinflammation, and improved aging memory disorder	(Kim et al., 2022)
	*Bifidobacterium dentium*	Chemical pathways	Stimulated intestinal chromaffin cells to secrete 5-HT and increased the expression of 5-HT receptor in hippocampus	(Engevik et al., 2021)

"NA" is "Not Applicable".

### Chemical pathways of the microbiota-gut-brain axis

Gut microbiota metabolites can directly or indirectly regulate brain activity through neuroendocrine and other pathways. Bacterial metabolites such as SCFAs, bile acids, vitamins, and other substances have direct effects. SCFAs are the main products of dietary fiber fermentation by intestinal microorganisms. They are mainly composed of acetate, propionate, butyrate, etc.; after being absorbed by intestinal epithelial cells, they pass through the portal vein to the liver, and through the systemic circulation directly communicate with the CNS (Dalile et al., 2019). In this case, SCFAs produced by gut microbiota, such as *F. prausnitzii*, *Eubacterium rectale*, *Bacteroides*, act on the hippocampus through the BBB and regulate neurogenesis (Tan et al., 2019). As an important metabolite of gut microbiota, the activation of SCFAs and their receptors play an important role in the regulation of neurogenesis: Butyrate activates receptors GPR41 (FFAR3) and GPR43 (FFAR2), enhances pituitary growth hormone release, and promotes brain neurogenesis (Aberg, 2010; Miletta et al., 2014); Mice lacking FFAR2 (*Ffar2^-/-^*) showed microglial cell morphology, dysfunction and cellular defects. FFAR2 signaling was also found to be expressed on splenic Iba-1^+^ myeloid cells in the red marrow, thus SCFAs may interact with FFAR2 receptors in peripheral myeloid cells to maintain normal microglia function and may be involved in the regulation of neurogenesis (Erny et al., 2015); Intervention with butyrate (histone deacetylase inhibitor) reduced cell proliferation and significantly increased apoptotic cells. In contrast, activation of the butyrate receptor GPR41 (FFAR3) reduced the elevation of histone acetylation and prevented the anti-proliferative and pro-apoptotic effects of butyrate (Wu et al., 2012). At the same time, some studies directly show that SCFAs are involved in the regulation of neurogenesis, compared with the control group, SCFAs intervention promoted the proliferation of hNPCs, and changed the expression of genes related to neurogenesis, proliferation and apoptosis, such as neurogenesis related genes *Atr*, *Ndn*, proliferation related genes *Cdk2*, *E2f1, Vegfa*, and apoptosis related genes *Bcl2*, *Bid*, *Casp8*, *Fas* (Yang L. L. et al., 2020); of these, physiological concentrations of SCFAs (acetate: butyrate: propionate in a ratio of approximately 30:1:2) all increased the proliferation rate of hNPCs. At the same order of magnitude, butyrate exerted a better effect on neurogenesis (Yang L. L. et al., 2020). In addition, systemic injection of sodium butyrate up-regulated histone hyperacetylation in the brain and liver, upregulating brain-derived neurotrophic factor (BDNF), nerve growth factor, and glial cell, glial cell line-derived neurotrophic factor (GDNF) expression in mouse hippocampus after intracerebroventricular injection of sodium butyrate (Varela et al., 2015). Bill acids have played a unique role in influencing the activities of the central nervous system. Although bile acids may be synthesized locally in the brain, they are mostly absorbed from the systemic circulation. In the brain, cytochrome P450 46A1 (CYP46A1) is involved in cholesterol clearance (Lorbek et al., 2012), this provides raw materials for bile acid synthesis. While bile acids also are synthesized in the liver and secreted to the intestine. After being modified by the gut microbiota, they are transported to the liver through the portal vein. A small amount of bile acids enters the brain through the BBB through the systemic circulation (Monteiro-Cardoso et al., 2021). In this scenario, the gut microbiota influences neurogenesis *via* the bile acid metabolism pathway. Tauroursodeoxycholic acid promotes NSCs proliferation and differentiation in mouse SGZ, and regulates mitochondrial function and cell cycle signals (Xavier et al., 2014; Soares et al., 2018); this phenomenon may also be related to lipid metabolism (Fernandes et al., 2020). Tauroursodeoxycholic acid produced by modification of gut microbiota regulates NSCs differentiation after blood circulation into the brain (Song et al., 2017; Chen et al., 2020b). The gut microbiota can often provide the host with a source of vitamins, the most common are Vitamin K, Vitamin C, and Vitamin B (folic acid), which may also regulate neurogenesis (Steinert et al., 2020). Compared with the control group, *Notch1* and *Hes1* expressions were up-regulated and the *Mash1* expression was down-regulated after folic acid administration. Folic acid activates Notch signaling, promoting the proliferation of hippocampal NSCs (Zhang et al., 2008; Zhang et al., 2012). Warfarin, an antagonist of Vitamin K, induced the proliferation of SVZ cells, and Vitamin K significantly reversed this phenomenon after the intervention, which may be related to Vitamin K-dependent proteins (Gely-Pernot et al., 2012). Meanwhile, a Vitamin K derivative, methylnaphthoquinone, could also regulate neuronal differentiation of NPCs (Sakane et al., 2017). Vitamin C also appears to play a potential role in regulating neurogenesis, increasing the number of DCX-positive neuroblasts and BrdU^+^-NeuN^+^ cells in the hippocampal DG. This is possibly related to the upregulation of BDNF expression (Nam et al., 2019). The tryptophan in dietary proteins is metabolized by metabolic enzymes in the gut microbiota to produce indole, tryptamine, etc. across the intestinal tight junction, or induce enterochromaffin cells (ECCs), enteroendocrine L-cells, etc. to release a series of substances to affect host activities (Roager and Licht, 2018). Compared with the specific pathogen-free control mice group, the content of indole derivatives in the serum of the germ-free (GF) group decreased as did neurogenesis. From this, we can infer that indole has an effect on neurogenesis after crossing the BBB (Wei et al., 2021). Moreover, indole supplementation up-regulated postsynaptic density protein-95 (PSD-95) and synaptophysin (SYP) expression in the hippocampus of WT C57BL/6J mice, while activating the Wnt/β-catenin pathway and up-regulating its downstream targets *VEGF* and *Neurog2* (Wei et al., 2021). SCFAs, indoles, bile acids, and gut microbiota produce and secrete serotonin (5-HT) by affecting ECCs (Benninghoff et al., 2010; Morris et al., 2017; Legan et al., 2022); the 5-HT produced in the gut cannot directly cross the BBB (Legan et al., 2022). However, tryptophan can cross the BBB and serve to synthesize 5-HT in the brain, which stimulates the corresponding receptors in different neurogenic regions to induce proliferation and neurogenesis: 5-HT1A heteroreceptor are involved in DG and SVZ; 5-HT1B autoreceptors modulate the role of 5-HT in SVZ and DG, 5-HT1B heterotypic receptors regulate cell proliferation in SGZ; 5-HT2A and 5-HT2C receptors selectively regulate cell proliferation in SGZ and SVZ. (Banasr et al., 2004; Liu N. et al., 2021). Taken together, some gut microbiota metabolites which cross the BBB could potentially regulate CNS activity, including neurogenesis.

### Neuronal pathways involved in the microbiota-gut-brain axis

The neuronal connections of the MGB axis underlie the fastest and most direct brain-gut interaction; the gastrointestinal tract interacts with the brain through the autonomic nervous system, of which the vagus nervous system is the main driver (Bonaz et al., 2018; Morais et al., 2021). The vagus nerve descends through the enteric nervous system to the gut, and enteric ganglia and nerve fibers make up two major plexuses: the submucosal and the myenteric nerve plexis (Wang and Powley, 2007). As a second brain, enteroendocrine cells are the first-order neurons underlying brain-gut interactions (Kaelberer et al., 2018). Gut microbiota metabolites stimulate Enteroendocrine L-cell, ECCs to generate 5-HT, glucagon like peptide-1 (GLP-1), cholecystokinin and other substances, and transmit signals to the enteric nervous system (Bellono et al., 2017; Kuwahara et al., 2020). On the one hand, ECCs secreted peptides/hormones act directly on vagal afferent fibers (Latorre et al., 2016), and on the other hand, secretions from the ECCs communicate with the vagus nerve *via* the enteric nervous system, intrinsic primary afferent neurons (Kuwahara et al., 2020). The vagus nerve might be involved in neurogenesis as evidenced by the fact that compared with the control group, capsaicin treatment resulted in unmyelinated vagus nerve injury, significantly reducing the number of doublecortin-positive cells in the DG and activated microglia (Ronchi et al., 2012). In another study, transplantation of fecal microbiota from old mice into young mice reduced neurogenesis, a process that may be related to decreased vagal activity (Rei et al., 2022). This suggests that the neuronal pathways of MGB axis could be involved in the regulation of glia and neurogenesis. First, the vagus nerve stimulates microglia activation in pathological conditions. For instance, in AD, the number of microglia branches and total branch length were significantly different under non-invasive vagus nerve stimulation (Kaczmarczyk et al., 2017). At the same time, vagus nerve stimulation down-regulated Toll-like receptor 4 (TLR4) expression in microglia in the acute phase of stroke and promoted microglia polarization to the M2 phenotype (Zhang et al., 2021). In addition, the vagus nerve regulates BDNF content in the brain; after vagotomy, BDNF expression in the hippocampus is down-regulated and hippocampal cell proliferation, neonatal cell survival, and neurogenesis were reduced. Therefore, mouse gut microbiota could induce changes in hippocampal BDNF and affect neurogenesis through the vagal pathway (Bercik et al., 2011; O’Leary et al., 2018). *L. rhamnosus* (JB-1) feeding upregulates the receptors for γ-aminobutyric acid (GABA) *GABA_Aα2_* receptor expression in the DG and CA3 regions (Bravo et al., 2011), while simultaneously down-regulating *GABA_Aα1_* receptor expression in DG, CA3, and CA1; this phenomenon was reversed after sub-diaphragmatic vagotomy (Bravo et al., 2011). In general, GABA_A_ activation inhibits cell proliferation, affects migration, maturation and differentiation (Pallotto and Deprez, 2014). At the same time, the dorsal vagal complex of the adult brain stem is a neurogenic region (Bauer et al., 2005; Charrier et al., 2006), the vagus nerve perceives sensory stimulation from gut microbiota and transmits to this region to affect neurogenesis. For example, ghrelin regulates the secretion of growth hormone and energy balance of the dorsal motor nucleus of the vague, and promotes neuronal proliferation, but at present there is controversy about which way to mediate (Zhang et al., 2004; Perello et al., 2022). In addition, vagus nerve stimulation activates the nucleus tractus solitarii; preproglucagon neurons in the nucleus tractus solitarii are the main source of GLP-1 in the brain (Holt et al., 2019; Cooper et al., 2021). The GLP-1 analog (Val8) increases the number of BrdU^+^ cells in the DG of the hippocampus and DG neurogenesis compared to controls (Mcgovern et al., 2012), as did two other GLP-1 analogs, Exendin-4 and liraglutide (Hamilton et al., 2011). Therefore, the vagus nerve could modulate neurogenesis by affecting central GLP-1 levels.

### Immune pathways associated with the microbiota-gut-brain axis

The crosstalk between gut microbiota and immune system is the basis for the connections between the brain and microorganisms through immune pathways. The CNS and gut microbiota can interact through the immune system, which is also affected by the CNS and gut microbiota (Zheng et al., 2020). Some studies show that gut microbiota and metabolites affect brain microglia cells, which may further affect neurogenesis. Compared with the control group, the expression of activated genes such as *Mapk8* or *Fcgr2β* in the microglia of the GF mice group was down-regulated as was *B2m* gene expression (the MHC class I related β2 microglobulin) and the microglia tended to be immature M0 type (Erny et al., 2015), these reveals the defect of microglia in GF mice; but the conditions were reversed after colonization and SCFAs diet (Erny et al., 2015). Therefore, we think that gut microbe-derived SCFAs enter the systemic circulation and cross the BBB to regulate microglial activity (Huuskonen et al., 2004). The gut microbiota affects BBB permeability as shown by increased BBB permeability in GF mice relative to control mice (Braniste et al., 2014); the expression of occludin and claudin-5 in the GF frontal cortex, striatum and hippocampus was reduced and the number of intact tight junctions significantly reduced (Braniste et al., 2014). In addition, there are other potential mechanisms by which the microbiota regulates microglia and astrocytes, such as microbe-associated molecular patterns, vagus nerve pathways, etc. (Wang Y. et al., 2018). For example, intestinal microbial metabolites such as H_2_S stimulate the vagus nerve and regulate microglial polarization (Chen et al., 2022; Ni et al., 2022), which regulates NSCs proliferation and differentiation (Shigemoto-Mogami et al., 2014). In some cases, the peripheral immune system can affect the central immune system. Similarly, some peripheral cytokines may enter the CNS through an abnormal BBB, neural route, and immune cell infiltration (Beurel et al., 2020). Therefore, changes in the gut microbiota may modulate the central immune system and glial function and could therefore have various effects on neurogenesis, a hypothesis confirmed by some studies. Antibiotics decreased Ly6Chi monocyte populations and neurogenesis in the mouse brain and bone marrow. These effects were reversed by probiotic treatment (Mohle et al., 2016). This suggests that Ly6Chi monocytes play a mediate between gut microbiota and neurogenesis. IL-4-driven microglia in the CNS activates the BDNF-TrkB pathway to induce neurogenesis in the hippocampus (Zhang J. et al., 2021). IFN-γ injection into the ventricle increased density and area of mouse hippocampal glial cells, activated microglia-mediated neuroinflammation, increased TNF-α, iNOS, etc. (Zhang J. et al., 2020) while inhibiting neural stem/precursor cells (NSPCs) proliferation and promoted the apoptosis of immature neurons (Zhang J. et al., 2020). In addition, the microglial activation-derived miR-146a-5p downregulates kruppel-like factor 4(KLF4) and cyclin-dependentkinase-like 5(CDKL5) expression in the DG region of rats, inhibiting NSCs proliferation and differentiation (Fan et al., 2022). Normal mice transplanted with fecal bacteria from AD mice developed memory impairment and reduced neurogenesis in the hippocampus, while TNF-α and IL-1β increased BDNF expression was downregulation (Kim et al., 2021). Moreover, changes in gut microbiota of mice can lead to increased colitis (Kim et al., 2021), characterized by changes in gut microbiota, like increased abundance of *Verrucomicrobia* and *Proteobacteria*. In such a situation, IFN-γ, IL-1β, TNF-α were up-regulated in the distal colon, resulting in microglial activation in adulthood and impaired hippocampal neurogenesis (Salvo et al., 2020). This evidence suggests that alterations in the gut microbiota modulate CNS immunity and alter glial function, which may affect neurogenesis.

## Traditional Chinese Medicine can affect neurogenesis by regulating gut microbiota

**Figure 5:**
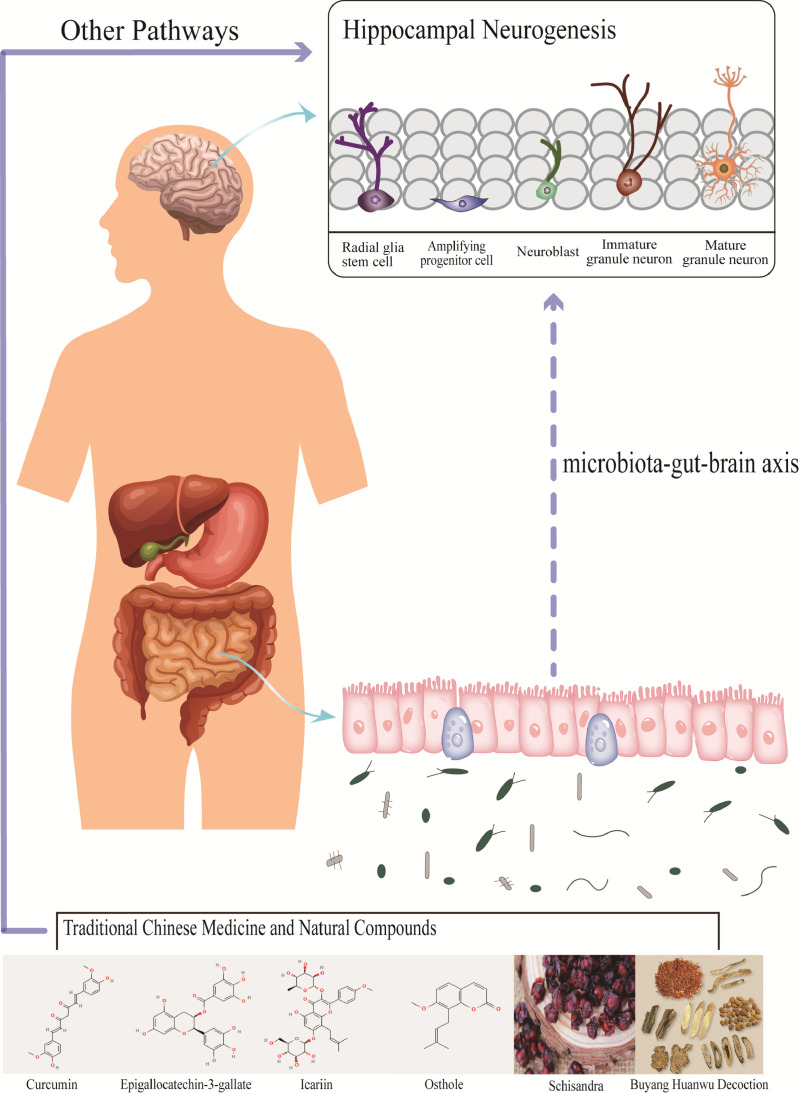
Traditional Chinese medicine and natural compounds affect the abundance of gut microbiota, and change the niche of neural stem cells through the three major pathways of microbiota-gut-brain axis, which may affect neurogenesis. However, there is little evidence that most Traditional Chinese medicine and natural compounds change neurogenesis through this pathway.

A potential association between dietary regulation and neurogenesis was found in some randomized controlled trials (Mohamed et al., 2013; Brandhorst et al., 2015; Ashton et al., 2019; Kim et al., 2020) suggests that external factors may regulate neurogenesis through gut microbiota. As TCM can be orally administrated, it inevitably interacts with the gut microbiota, improving TCM oral availability while TCM also impacts the gut microbiota and the MGB axis (Yang et al., 2014). Here we speculate that TCM can regulate neurogenesis under physiological or pathological conditions by remodeling the gut microbiota. Below, we summarize TCM’s effects on the most common types of bacteria in the human body: *Bacteroidetes*, *Firmicutes*, *Proteobacteria*, and *Actinobacteria* (**Figure 5** and **Table 3**).

**Table 3:** Effects of Traditional Chinese Medicine and Compound on Gut Microflora and Neurogenesis.

Herbal Formula/Compound	Disease	Changes in the composition of gut microbiota	Neurogenesis	Reference
Curcumin	Alzheimer’s Disease	Family: *Bacteroidaceae, Prevotellaceae, Lactobacillaceae*, *Rikenellaceae* Genus: *Prevotella, Bacteroides, Parabacteroides, Escherichia/Shigella*	Promoted endogenous NSCs proliferation and neurogenesis, activate the Notch pathway	(Sun et al., 2020) (Li et al., 2019)
Epigallocatechin-3-gallate	Parkinson’s disease	Phylum: *Firmicutes, Bacteroidetes* Genus: *Acetobacter, Lactobacillus*	Promoted hippocampal NPCs proliferation and neurogenesis, activate the SHH pathway	(Wang et al., 2012) (Xu et al., 2020)
Icariin	Alzheimer’s Disease	Genus: *Lactobacillus, Bifidobacterium, Adlercreutzia, Bacteroides, Paraprevotella*	Promoted NSCs proliferation, migration, and differentiation	(Ma et al., 2021) (Zhang T. et al., 2020)
Osthole	Neuropathic Pain	Genus: *Akkermansia, Bacteroides, Lachnospiraceae_unclassified, Lachnospiraceae_NK4A136_group*	Promoted NSCs proliferation, activate the Notch pathway	(Li R. et al., 2022) (Kong et al., 2015)
Gastrodin	Alzheimer’s Disease	Phylum: *Firmicutes, Verrucomicrobia, Bacteroidetes* Class: *Clostridia, Verrucomicrobiae, Firmicutes, Bacteroidia, Negativicuts*	Promoted NPCs proliferation and differentiation, activate the MAPK pathway	(Li and Qian, 2016) (Fasina et al., 2022)
Ginsenoside Rg1	Alzheimer’s Disease	Phylum: *Proteobacteria, Firmicutes* Family: *Enterobacteriaceae, Streptococcaceae, Pasteurellaceae* Genus: *Lactobacillus, Escherichia-Shigella*	Promoted hUCMSCs proliferation, differentiate into NSCs, downregulated the Wnt/β-catenin, Notch pathway	(Wang L. et al., 2020) (Xiao et al., 2022)
Berberine	Parkinson’s disease	Genus: *Enterococcus, Escherichia–Shigella, Pseudomonas, Lactobacillus*	Promoted NSCs survival, differentiation, upregulated the Wnt/β-catenin pathway	(Shou et al., 2019) (Wang Y. et al., 2021)
Quercetin	Alzheimer’s Disease	Phylum: *Epsilonbacteraeota*	Promoted NSCs/NPCs proliferation, differentiation	(Yang S. et al., 2020) (Karimipour et al., 2019)
Resveratrol	Alzheimer’s Disease	*p_Bacteroidetes, c_Bacteroidia, o_Bacteroidales, f_Muribaculaceae, g-norank_f_Muribaculaceae*	Promoted the implantation of hUC-MSCs in the hippocampus, promoted hippocampal neurogenesis, inhibited apoptosis of hippocampal neurons	(Wang X. et al., 2018) (Li et al., 2021)
Ginkgolide B	Alzheimer’s Disease	Phylum: *Firmicutes, Bacteroidota* Order: *Lactobacillales, Bacteroidales* Family: *Muribacullaceae, Lactobacillaceae* Genus: *Lactobacillus*, *Alloprevotella*	Promoted NSCs differentiation, activate Wnt/β-catenin pathway	(Liu J. et al., 2021) (Li et al., 2018)
Baicalin	Cerebral ischemia-reperfusion	Species: *Halomonas_smyrnensis, Parabacteroides_johnsonii, Bacteroides_uniformis, Citromicrobium_sp_WPS32, Eubacterium_sp_CAG_86, Lactobacillus_plantarum*	Promoted hippocampal DG proliferation, differentiation	(Liu J. et al., 2020) (Zhuang et al., 2013)
Puerarin	Depression	Phylum: *Firmicutes, Bacteroidetes, Proteobacteria, Actinobacteria* Class: *Bacilli/Clostridia, Actinobacteria* Order: *Bacteroidales, Campylobacterzles, Desulfovibrionales, Lactobacillales, Bacillales*	Activated ERK1/2, PI3K/Akt, Nrf2/HO-1 pathway enhances NGF induced neurogenesis	(Song et al., 2021) (Zhao et al., 2015)
Salidroside	Alzheimer’s Disease	Phylum: *Bacteroidetes/Firmicutes*, *Chloroflexi* Genus: *Norank_f_Muribaculaceae, Alloprevotella, Parasutterella, Lachnospiraceae_NK4A136_group, Unclassified_f_Lachnospiraceae, Alistipes,Norank_f_Lachnospiraceae, Odoribacter, Rikenellaceae_RC9_gut_group, Ruminococcaceae_UCG-014, Ruminiclostridium_9*	Inhibited MTC proliferation, promoted MTC neural differentiation, negatively regulated Notch pathway, positively regulated BMP pathway	(Zhao et al., 2014) (Xie et al., 2020)
Paeoniflorin	Depression	Genera: *Lactobacillus, Lachnospira, Eubacterium_ruminantium_group, Roseburia, Facklamia, Jeotgalicoccus, Ruminiclostridium_5, Eubacterium_coprostanoligenes_group*	Promoted hippocampal NSCs proliferation, differentiation, activated the BDNF-TrkB pathway	(Yu et al., 2019) (Chen L B. et al., 2019)
Andrographolide	Gulf War Illness	Phylum: *Firmicutes, Bacteroidetes* Genus: *Lachnospiraceae_ug, Akkermansia, Bifidobacterium, Staphylococcus*	Promoted hippocampal NPCs proliferation, activated the Wnt/β- catenin pathway	(Saha et al., 2021) (Varela-Nallar et al., 2015)
Schisandra	Depression	Phylum: *Bacteroidetes, Firmicutes* Genus: *Bacteroides, Lactobacillus*	Promoted DG region proliferation, differentiation, maturation	(Cai et al., 2020) (Yan et al., 2021)
Buyang Huanwu Decoction	Cerebral ischemia	Phylum: *Actinobacteria, Deferribacteres, Dependentiae* Family: *Arcobacteraceae, Vibrionaceae, Enterobacteriaceae* Genus: *Escherichia-Shigella, Klebsiella, Streptococcus, Coprococcus_2, Enterococcus, Lactobacillus, Faecalibacterium, Ruminococcaceae_UCG-002*	Promoted hippocampal neurons proliferation, maturation	(Tang R. et al., 2022)
Liuwei Dihuang Decoction	Alzheimer’s disease	Genus: *Adlercreutzia, Anaerotruncus, Ruminococcus, Prevotella, Streptococcus, Veillonella, Bilophila, Coprococcus*	Promoted DG neurogenesis	(Lee et al., 2005) (Wang J. et al., 2016)
Xiaoyaosan	Depression	Phylum: *Bacteroidetes, Proteobacteria, Firmicutes, Chloroflexi, Planctomycetes*	Promote DG nerve survival, upregulate BDNF expression	(Gao L. et al., 2018) (Zhu et al., 2019)

### Bacteroidetes

*Bacteroidetes* maintain the balance of gut microbiota and are closely related to CNS diseases. For example, increased *Bacteroidetes* levels were found in patients with AD, major depressive disorder, and myasthenia gravis (Jiang et al., 2015; Vogt et al., 2017; Moris et al., 2018), and decreased levels in patients with PD (Unger et al., 2016). At the same time, changes in *Bacteroidetes* abundance are accompanied by changes in microglia, astrocytes, etc. (Shi et al., 2021). This suggests that *Bacteroidetes* may regulate NSCs and neurogenesis. Curcumin treatment corrected the relative abundance of *Bacteroidetes* in a mouse anxiety model, increasing SCFAs content in the feces, affecting the glycerophospholipid metabolism in the prefrontal cortex, possibly by regulating circulatory pathways (Zhang et al., 2022). In another study, cAMP-response element binding protein activation enhanced BDNF expression by increasing glycerophospholipids (Hossain et al., 2022). Meanwhile, Curcumin intervention up-regulated 5-HT(1a) receptor and BDNF, increasing neurogenesis (Xu et al., 2007). This suggests that curcumin may modulate neurogenesis by affecting chemical pathways in the MGB axis by modulating *Bacteroidetes* activity. In addition, *Scutellaria baicalensis Georgi* also exerts similar pharmacological effects in brain injuries induced by diabetes, mice treated with Astragalus membranaceus reduced *Bacteroidete*s abundance, upregulation of BDNF expression and promotion of hippocampal mitogenesis (Li X. et al., 2022). Baicalin, an effective component of *Scutellaria baicalensis Georgi*, reduces the abundance of *parabacteroides*, *prevotella*, and *Bacteroides* in mouse intestine, reducing the proinflammatory factors IL-1β, IL-6 and TNF-α in the cerebral cortex (Gao et al., 2018b); in the DG, it promotes cell proliferation and differentiation, and upregulates hippocampal Wnt/β-catenin signaling pathway related protein expression (Xiao et al., 2021). This suggests that the gut microbiota may influence neurogenesis by affecting brain inflammation. Similarly, Astragalus and its active components affect neurogenesis through MGB axis immune pathways, which may be related to microglia activation (Li et al., 2020), but the specific mechanism needs to be further confirmed. *In vitro*, Astragaloside VI promotes the differentiation of primary NSCs into neurons and astrocytes, similarly to Astragalus flavor, which up-regulated *Notch1*, *Jagged1*, *Mash1*, *Ngn1*, and *Ngn2* expression in NSCs (Gao et al., 2022). Epigallo-catechin-3-gallate (EGCG), the main component of Camellia sinensis (green tea), increases the abundance of *Bacteroides uniformis*, *Bacteroides vulgatus*, *Bacteroides stercoris*, *Bacteroides thetaiotaomicron*, and *Bacteroides cellulosilyticus* in human gut microbiota (Liu Z. et al., 2020). In preclinical studies, EGCG enhanced hippocampal neurogenesis, improved learning and memory in mice, and upregulated SHH, Ptch and Gli1 expression in the mouse hippocampus (Wang et al., 2012). Crossing the intestinal epithelial barrier and the BBB regulates hippocampal neural differentiation after EGCG interacts with the gut microbiota (Faria et al., 2011; Granja et al., 2019). Qisheng Wan formula increased *Bacteroidetes* abundance in AD mice while reducing NF-κB, IL-6, and TNF-α content (Xiong et al., 2022); one of its active ingredients, *Polygala tenuifolia*, promotes APP-NSCs proliferation and migration and neuronal differentiation (Wang X. F. et al., 2021), while another ingredient, *Acorus tatarinowii Schott*, also affects neurogenesis, microglial function and can activate PKA-CREB signaling, enhancing nerve growth factor induced differentiation and neurite length of PC12 cells (Cai et al., 2016; Lam et al., 2016). Lipopolysaccharide, amyloids, and other substances produced by *Bacteroidetes* affect neuroinflammation, possibly by regulating the brain inflammatory microenvironment to regulate neurogenesis (Zhao and Lukiw, 2018), as confirmed by the effects of TCM presented above. In summary, most TCM compounds regulate neurogenesis by interfering with the chemical and immune pathways of the MGB axis, accompanied by changes in *Bacteroidetes* abundance.

### Firmicutes

*Firmicutes* transplantation can be used in the treatment of brain diseases. *Lactobacillus*, as a unique *Firmicutes* of bacteria, is one of the important microbiota for maintaining the micro-ecological health of the human gut, some studies have found increased 5-HT and BDNF concentration in the serum of constipated patients who received *Lactobacillus reuteri DSM-17938* (Riezzo et al., 2019), *Lactobacillus rhamnosus HN001* could effectively prevent and treat postpartum depression (Slykerman et al., 2017). This evidence indicates that *Firmicutes* may regulate brain activity and treatment of central nervous system diseases and injuries. Using a Buyang Huanwu Decoction (BHD) in cerebellar ischemia model mice significantly down-regulated the relative abundance of *Streptococcus*, *Coprococcus_2*, *Enterococcus*, etc, and while up-regulating the relative abundance of *Lactobacillus*, *Ruminococcaceae_UCG-002*, etc. (Tang R. et al., 2022). This lowered the levels of proinflammatory cytokines in peripheral blood and inhibited microglial activation (Tang R. et al., 2022). At the same time, BHD promoted NSCs proliferation in middle cerebellar artery occlusion mice and promoted astrocyte and neuronal differentiation, which may be related to Jak/Stat3/Cyclin D1 and EGFR/PI3K/Akt/Bad/14-3-3 pathways (Chen X. et al., 2020). The altered abundance of Firmicutes after BHD intervention may affect peripheral and central immunity, thus regulating NSCs proliferation and differentiation. In addition, curcumin promoted hippocampal neurogenesis and enhanced NSCs proliferation in APP/PS1 mice, up-regulating Hes1 and NICD expression and activating the Notch pathway (Li et al., 2019). In the gut of APP/PS1 mice, curcumin down-regulated the abundance of *Lactobacillaceae*, producing substances such as demethylcurcumin and bisdemethoxycurcumin which alleviate the pathological changes of AD (Sun et al., 2020). Changes in the gut microbiota may modulate gene expression in neurogenesis-related pathways; however, there is currently no clear evidence for the specific mechanisms. In some mental diseases, curcumin increases the content of 5-HT, BDNF, etc. in the hippocampus, and modulates related signaling changes in the peripheral intestinal system (Yu et al., 2015); this may be related to the hypothalamic-pituitary-adrenal (HPA) axis of the MGB axis, as curcumin reduces the ratio of adrenal gland weight to body weight and adrenal cortex thickness, down-regulating serum corticosterone levels and up-regulating hippocampal glucocorticoid receptor expression (Xu et al., 2006). In the SGZ, andrographolide promotes NSCs proliferation and neurogenesis, up-regulating hippocampal β-catenin expression, inhibiting GSK-3β activity, and up-regulating the expression of Wnt target gene *NeuroD1* (Varela-Nallar et al., 2015). Moreover, Andrographolide increased the abundance of *Firmicutes* in the gut, significantly reducing neuroinflammation in mouse brain tissue and enhancing BDNF expression (Saha et al., 2021). This suggests that Andrographolide may affect BDNF expression through the MGB axis, affecting Wnt signaling in NSCs (Yang et al., 2016; Li et al., 2017), and may be involved in neuroinflammation. Increased abundance of intestinal *Firmicutes* in AD mice after Ginkgolide B treatment could improve AD pathology and cognitive dysfunction (Liu J. et al., 2021). *In vitro*, Ginkgolide B promotes NSCs neuronal differentiation in the SVZ, activates the Wnt/β-catenin pathway, and upregulates the target gene *Axin2* (Li et al., 2018). This may be related to the *Firmicutes* regulating the permeability of the BBB and promoting Ginkgolide B to cross the BBB to regulate hippocampal activity (Lin et al., 2022). In addition, Resveratrol packaged with selenium nanomaterials (TGN-Res@SeNPs) reversed the alterations of *Firmicutes* in AD, increasing *Lactobacillus*, *Lachnospiraceae_NK4A136_group*, etc (Li et al., 2021); after treatment, levels of neurotransmitters such as glutamate and GABA recovered in the hippocampus of mice, increased total antioxidant capacity (T-AOC), CAT, GSH-Px, and IL-10 levels in mouse brain tissue and decreased MDA, IL-1β, IL-6, TNF-α (Li et al., 2021). This may be because Resveratrol modulates the abundance of *Firmicutes*, such as *Lachnospiraceae_NK4A136_group*, which are closely related to inflammation and oxidation and may affect neurogenesis by altering the microenvironment (Li and Barres, 2018). A high-fat diet can damage cognitive function, which may be related to neuroinflammation (Huang and Yun, 2020). When high-fat diet mice were treated with a water extract from a processed Polygonum multiflorum modulate, a significant decrease in the relative abundance of *Firmicutes* was observed (Gu et al., 2020); in addition, Polygonum multiflorum Thunberg complex orally Composition-12 (PMC-12) promoted cell proliferation and neuronal differentiation in the DG and significantly up-regulated BDNF and p-CREB expression in the hippocampus. However, the underlying mechanism by which Polygonum multiflorum affects the MGB axis remains unclear (Park et al., 2016).

### Proteobacteria

Changes in *Proteobacteria* can predict changes in the total brain and cortex of rhesus monkeys in infancy (Rendina et al., 2021). *Proteobacteria* levels differ between healthy individuals, and those with mild cognitive impairment and AD; these difference gradually increase with disease deterioration (Guo et al., 2020). The density of DCX-positive cells in the DG of *Bilophila wadsworthia* (Belongs to *Proteobacteria*) colonized mice decreased, showing a similar effect on mouse hippocampus to that of a ketogenic diet, affecting hippocampal neurogenesis (Olson et al., 2021). Berberine, a TCM compound, could increase the abundance of dopa/dopamine producing bacteria such as *Escherichia-Shigella* and *Pseudomonas* (Wang Y. et al., 2021). Berberine neuronal differentiation of C17.2 NSCs *in vitro*, upregulates Nestin, microtubule-associated protein 2 (MAP2) and tubulin β 3 Class III (TUBB3) expression, this indicates that the proliferation and neuronal differentiation of NSCs are promoted after the intervention. This may be closely related to the activation of Wnt/β-catenin pathway (Shou et al., 2019). Berberine might affect central dopamine production by regulating *Proteobacteria* abundance, further affecting Wnt/β-catenin through Dopamine D2 receptor to promote neurogenesis in PD (Marchetti et al., 2022). Ginsenoside Rg1 was shown to reduce tau content in the hippocampus of AD model and upregulated *Proteobacteria* abundance (Wang S. et al., 2020), promoting NSCs growth and proliferation, down-regulating β-catenin, C-myc expression and upregulating GSK-3β expression (Xiang et al., 2019). This may be caused by changes in bacterial tryptophan metabolism, reducing peripheral blood 5-HT levels and affecting hippocampal neurotransmitters, thereby regulating NSCs proliferation and differentiation (Chen et al., 2022); however, the specific mechanism warrants further research. Similarly, BHD decreased the abundance of *Escherichia-Shigella* and *Klebsiella* (Tang R. et al., 2022). The combination of *Puerariae Lobatae Radix* (PLR) and *Chuanxiong Rhizoma* (CXR) could partially reduce the enrichment of intestinal pathogens *Escherichia_Shigella*, *Proteus*, *Klebsiella*, etc. in a mouse model of ischemic stroke (Chen R. et al., 2019). Plasma fluorescein isothiocyanate-dextran concentrations and intestinal permeability decreased, and claudin-5 and ZO-1 levels in the brain and colon returned to normal levels after this treatment. This suggests that PLR and CXR can prevent brain gut-barrier disruption caused by ischemic stroke (Chen R. et al., 2019). Moreover, *Rhizome Chuanxiong* and the other three TCM compounds could promote NSCs proliferation *in vitro*, and also promote the proliferation of hippocampus *in vivo*, while inhibiting hyperactivity of the HPA axis (Pao et al., 2012). This suggests that the Chuanxiong may affect neurogenesis through the gut microbiota and HPA axis, but this needs further confirmation.

### Actinobacteria

*Actinobacteria* levels are low in the human intestine, but they are extremely important to maintain intestinal homeostasis. Differences in *Actinobacteria* emerge early in infancy, arising particularly between cesarean and vaginal births (Gronlund et al., 1999; Kabeerdoss et al., 2013). In a clinical trial, transplantation of *Bifidobacterium longum NCC3001* of *Actinobacteria* improved depressive manifestations and improved quality of life in patients with irritable bowel syndrome, which was associated with altered amygdala and frontal limbic activity (Pinto-Sanchez et al., 2017). This suggests that *Actinobacteria* could affect neurogenesis. Ginsenoside Rg3, one of the main components of red ginseng, is fermented and metabolized to ginsenoside Rd in the intestine by *Bifidobacterium*. After treatment with ginsenoside Rg3, serum corticosterone and adrenal cortical hormones were reduced. In the brain, 5-HT was up-regulated and norepinephrine content was reduced, leading to increased expression of central BDNF (Han et al., 2020; Sur and Lee, 2022). Simultaneously, ginsenosides Rd promoted the proliferation of hippocampal NSCs but Andrographolide but did not affect differentiation (Lin et al., 2012). Therefore, we speculate that red ginseng regulates central neurotransmitters and BDNF through the HPA axis under the action of intestinal *Actinobacteria*, which further affects NSCs differentiation. Paeoniflorin modulates gut microbiota in depressive rats, upregulating *Roseburia* abundance, and has antidepressant effects; the gut microbiota metabolizes paeoniflorin to produce benzoic acid, which can cross the BBB to regulate the CNS (Yu et al., 2019). Paeoniflorin up-regulated BDNF expression, enhanced NSCs proliferation in the hippocampus and promoted astrocyte differentiation (Chen L B. et al., 2019). Sodium benzoate can increase BDNF expression in neurons in a dose-dependent and time-dependent manner (Jana et al., 2013; Guo et al., 2020). This suggests that the benzoic acid produced by *Actinobacteria* metabolization of paeoniflorin may directly regulate neurogenesis. At present, there are few studies on the effects of TCM on *Actinobacteria*, but their role in regulating neurogenesis warrants further research.

## Conclusions and future prospects

Accumulating evidence suggests that impaired neurogenesis exist in pathological states of the CNS (Koh and Park, 2017; Berger et al., 2020). Neurogenesis, including NSCs growth, development, and differentiation in the hippocampus is tightly regulated by Notch, Wnt, and the extracellular matrix. The regulation of these pathways is affected by external factors, so it can regulate neurogenesis by regulating external stimuli. Based on this theory, gut microbiota plays a potential role in neurogenesis.

Some studies have found that the hippocampus is sensitive to diet (Hueston et al., 2017; Davidson and Stevenson, 2022); since changes in dietary habits and nutrition directly affect gut microbiota activity, this may in turn affect hippocampal activity. TCM is often taken orally through the digestive system. Accordingly, TCM could modulate hippocampal activity through the gut microbiota.

Numerous clinical and preclinical studies provide robust data on the microbiome. The MGB axis allows gut-brain interactions: the gut microbiota regulates CNS development and function through neural, immune, metabolic, and other pathways through the MGB axis. The way of administration of TCM plays a huge advantage in regulating gut microbiota. Therefore, we speculate that TCM regulates gut microbiota abundance, affects MGB axis, and further exerts the pharmacological effect of CNS.

In this review, we highlighted the role of gut microbiota on neurogenesis through the MGB axis and summarized the potential of TCM for modulating the CNS through this pathway. Even if the relevant mechanism has not been fully and systematically investigated, existing data have closely linked both phenomena. The connection between neurogenesis and the gastrointestinal tract may lead to strategies to treat CNS diseases by targeting gut-brain interactions. In addition, the connection between neural activity and systemic metabolism also provides a new research direction for neurogenesis. In-depth research on this topic not only provides suggestions and guidelines for TCM therapy for some neurological diseases, but also improves the theory of neurodevelopment.

## Author contributions

CZ, PX and XL provided the writing of articles. HuiZ, CT, SZ organized tables and figures, HaiZ, WL, and LS revised the article and put forward key suggestions. JW, BZ and WL provided financial support. All authors contributed to the article and approved the submitted version.
